# Low early complication rates after arthroscopic meniscus repair and meniscectomy

**DOI:** 10.1007/s00167-023-07507-8

**Published:** 2023-07-14

**Authors:** Wilson C. Lai, Tyler R. Mange, Theofilos Karasavvidis, Yu-Po Lee, Dean Wang

**Affiliations:** 1 Department of Orthopaedic Surgery, UCI Health, 101 The City Drive S. Pavilion III, 2nd Floor, Orange, CA 92868 USA; 2 Department of Biomedical Engineering, University of California Irvine, Irvine, CA USA

**Keywords:** Meniscus repair, Meniscectomy, Arthroscopy, Complications, Pulmonary embolism, Infection, Readmission, Reoperation

## Abstract

**Purpose:**

To evaluate the 30-day complication rates after arthroscopic meniscus repair and meniscectomy using the American College of Surgeons National Surgical Quality Improvement Program (ACS-NSQIP) database, with subgroup analysis of patients aged > 40 years.

**Methods:**

NSQIP registries between 2006 and 2019 were queried using Current Procedural Terminology codes to identify patients undergoing arthroscopic meniscus repair (CPT 29882, 29883) and meniscectomy (29880, 29881). The following 30-day complications were assessed: pulmonary embolism (PE), venous thromboembolism (VTE), surgical site infection (SSI), reoperation, and readmission. Complications rates between treatment groups were compared using multivariate logistic regression analyses adjusted for sex, age, steroid use, and smoking/dyspnoea/COPD. A subgroup analysis was performed for patients aged > 40 years.

**Results:**

A total 6354 meniscus repairs and 99,372 meniscectomies were identified. Complication rates were < 1% for both meniscus repair and meniscectomy. Meniscus repair was associated with significantly higher rates of PE, VTE, and readmission compared to meniscectomy: PE (0.2% vs 0.1%, *p* < 0.001), VTE (0.8% vs 0.4%, *p* < 0.001), superficial SSI (0.1% vs 0.2%, n.s), deep SSI (0.07% vs 0.1%, n.s), reoperation (0.5% vs 0.4%, n.s), and readmission (0.9% vs 0.8%, *p* = 0.003). Among patients aged > 40 years, complication rates were < 1.3% for both meniscus repair and meniscectomy. Similar trends and rates were found in patients aged > 40 years undergoing meniscus repair versus meniscectomy: PE (0.38% vs 0.12%, *p* < 0.001), VTE (1.07% vs 0.46%, *p* < 0.001), superficial SSI (0.03% vs 0.19%, n.s), deep SSI (0.1% vs 0.06%, n.s), reoperation (0.48% vs 0.43%, n.s), and readmission (1.2% vs 0.85%, *p* = 0.01).

**Conclusion:**

Arthroscopic meniscus repair and meniscectomy are both low-risk procedures with 30-day complication rates < 1% overall and < 1.3% among patients aged > 40 years. These findings support meniscus repair whenever feasible in the setting of preserved articular cartilage. Understanding of the short-term complication rates after arthroscopic meniscus repair and meniscectomy can aid surgeons in providing comprehensive preoperative counselling to patients considering such treatments, specifically when discussing the risks and benefits of meniscus repair.

**Level of evidence:**

III.

## Introduction

With recent advances in arthroscopic repair techniques, there have been increased efforts to repair meniscus tears whenever possible to preserve meniscus tissue and optimize tibiofemoral contact pressures [1]. Parker et al. showed a 37% increase in meniscal repair procedures and a 17% decrease in meniscectomy procedures over a recent 9-year period [18]. Multiple biomechanical studies have demonstrated that meniscal repair, unlike partial meniscectomy, restores load transmission to levels near those of an intact meniscus [4, 8, 11, 15, 16]. As a result, significantly improved patient-reported outcomes, higher rate of return to sport, and decreased progression to osteoarthritis after meniscal repair have been reported [6, 9, 19, 23].

Despite the increased trend in arthroscopic meniscal repair compared to meniscectomy, few studies have examined the short-term complications comparing the two procedures, particularly for patients over 40 years of age. Prior studies have demonstrated an increased risk of post-operative complications such as DVT and reoperation in patients 40 years of age or older shortly following arthroscopic knee surgery [13, 17]. This specific age group is particularly important as meniscus tears are more common in patients older than 40 years of age, and over 50% of meniscectomies are performed in patients 45 years of age or older [7].

Because the repairability of a meniscus tear is not always certain prior to surgery, and because post-operative rehabilitation can differ substantially between meniscus repair and partial meniscectomy, understanding of the short-term complication rates after these procedures can ultimately aid clinicians in the treatment of meniscus tears and in counselling patients regarding early post-operative risks. Thus, the purpose of this study was to evaluate the 30-day complication rates after arthroscopic meniscus repair and meniscectomy using the American College of Surgeons National Surgical Quality Improvement Program (ACS-NSQIP) database. The secondary aims of this study were to compare complication rates between arthroscopic meniscus repair and meniscectomy, with a subgroup analysis of patients aged > 40 years. The hypothesis was that both arthroscopic meniscus repair and meniscectomy would have low rates of 30-day post-operative complications, including pulmonary embolism (PE), venous thromboembolism (VTE), surgical site infection (SSI), reoperation, and all-cause readmission, in all age groups.

## Material and methods

This study was exempt from institutional review board approval due to deidentification of patient and surgeon information in the database. Data were retrospectively collected from the American College of Surgeons National Quality Improvement Program (ACS-NSQIP). The ACS-NSQIP is a prospectively collected, risk-adjusted, outcomes-based program with over 500 participating institutions in the USA. The 2019 version of the database was used, which contains more than 300 variables including preoperative risk factors, intraoperative variables, and 30-day post-operative complications for patients undergoing major surgical procedures. The database is maintained and updated by trained clinical reviewers, who extract patient information from patient interviews, medical records, and operative reports through the 30th post-operative day, regardless of discharge date [5]. The study was conducted according to the Strengthening the Reporting of Observational Studies in Epidemiology (STROBE) guidelines [24].

**Fig. 1:**
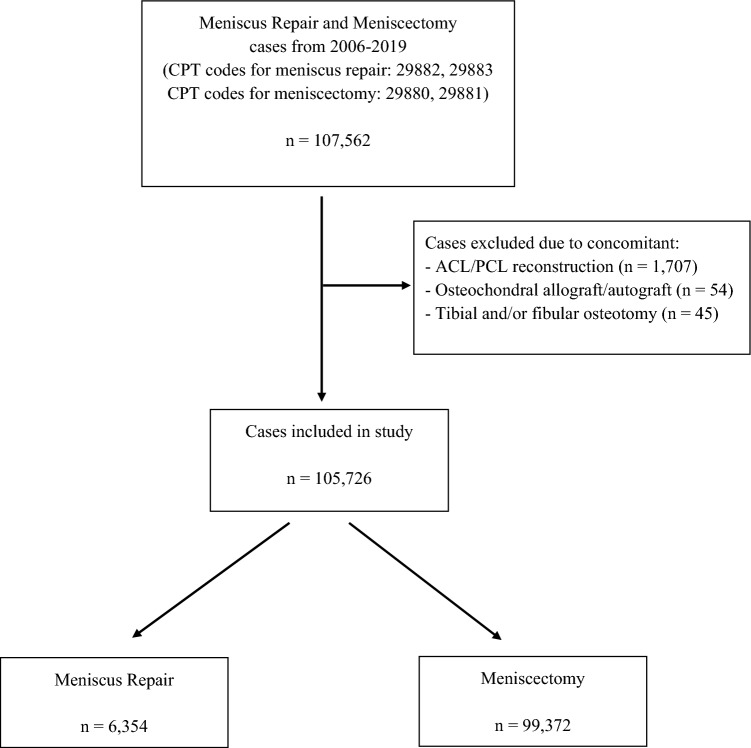
Flowchart for meniscus repair and meniscectomy cases. Flowchart demonstrating cases excluded due to concomitant procedures such as ACL/PCL reconstruction, osteochondral allograft/autograft transplantation, and osteotomy procedures

NSQIP registries between 1 January 2006 and 31 December 2019 were queried using Current Procedural Terminology (CPT) codes to identify patients undergoing arthroscopic meniscus repair (29882, 29883) and meniscectomy (29880, 29881). Cases with concomitant procedures that could potentially affect complication rates, including ligament repair or reconstruction procedures, osteotomies, and cartilage restoration procedures, were excluded (Fig. 1). Cases with minor concomitant arthroscopic procedures, such as chondroplasty, removal of loose/foreign body, and lateral release, were included in the analysis and concomitant procedures recorded. Patient demographics, including gender, age, body mass index (BMI), and history of comorbidities, including diabetes, smoking, dyspnoea, chronic obstructive pulmonary disease (COPD), functional status, congestive heart failure (CHF), hypertension (HTN), renal failure/dialysis, steroid use, and bleeding disorders were extracted for analysis. Operative time was also collected. In the NSQIP database, operative time is defined as the total operation time in minutes. The 30-day complications of interest included: pulmonary embolism (PE), total venous thromboembolism (VTE), wound disruption, superficial surgical site infection (SSI), deep SSI, reoperation, and all-cause readmission. VTE was defined as an event of DVT and/or PE.

### Statistical analysis

Continuous variables were described with mean ± standard deviation, whereas categorical variables were reported with absolute and relative frequencies. A t test was conducted to compare continuous variables, while binary outcomes were compared using the Chi-square or Fisher exact test as appropriate. Univariate and multivariate logistic regression models were developed to compare complication rates between meniscus repair and meniscectomy. The following variables were decided a priori to be included in the multivariate model: sex, age, steroid use, and respiratory status (smoking/dyspnoea/COPD). A subgroup analysis comparing complication rates between treatment groups was performed for patients aged > 40 years. The threshold for statistical significance was *p* < 0.05. For univariate and multivariate analyses for the entire cohort and patients > 40 years of age, a Bonferroni adjusted significance level of 0.0125 was calculated to account for the increased possibility of type I error with multiple comparisons. Stata 17 (StataCorp LLC, College Station, TX) was used as statistical software for all analyses.

## Results

After applying the inclusion and exclusion criteria, a total of 6354 meniscus repair and 99,372 meniscectomy procedures were identified and included in the final analysis. The number of concomitant procedures included in each group can be found in Table 1. Baseline patient demographics and pre-existing comorbidities are presented in Table 2.

**Table 1:** Concomitant surgical procedures

Concomitant surgical procedures	Meniscus repair (*n* = 6354) *n* (%)	Meniscectomy (*n* = 99,372) *n* (%)
Chondroplasty or multiple drilling or microfracture	395 (6.2)	4094 (4.1)
Lysis of adhesions	7 (0.1)	125 (0.1)
Removal of loose/foreign body	35 (0.6)	684 (0.7)
Lateral release	17 (0.3)	822 (0.8)
Other/unlisted procedure	562 (8.8)	3502 (3.5)
Total	1016 (16)	9227 (9.3)

**Table 2:** Baseline demographic and clinical characteristics

Variable	Meniscus repair (*n* = 6354) *n* (%)	Meniscectomy (*n* = 99,372) *n* (%)	*p* value
Female	2362 (37.2%)	45,556 (45.9%)	< 0.001
Age (mean, SD)	40.2 ± 15.7	52.6 ± 13.9	< 0.001
Age ≤ 40 years	3451 (54.3%)	18,039 (18.2%)	< 0.001
BMI (mean, SD)	29.7 ± 6.4	31.7 ± 7.3	(n.s)
Steroid use	53 (0.8%)	1417 (1.4%)	< 0.001
Diabetes	376 (5.9%)	10,645 (10.7%)	< 0.001
Smoking	1044 (16.4%)	15,108 (15.2%)	
Dyspnoea	88 (1.4%)	2683 (2.7%)	< 0.001
Functional health status prior surgery			(n.s)
Independent	6290 (99.0%)	98,046 (98.7%)	
Partially dependent/dependent	35 (0.6%)	362 (0.4%)	
COPD	56 (0.9%)	1870 (1.9%)	< 0.001
CHF in 30 days before surgery	2 (0.0%)	96 (0.1%)	(n.s)
HTN	1119 (17.6%)	35,499 (35.7%)	< 0.001
Renal failure/dialysis	2 (0.0%)	118 (0.1%)	(n.s)
Bleeding disorders	33 (0.5%)	1085 (1.1%)	< 0.001

**Fig. 2:**
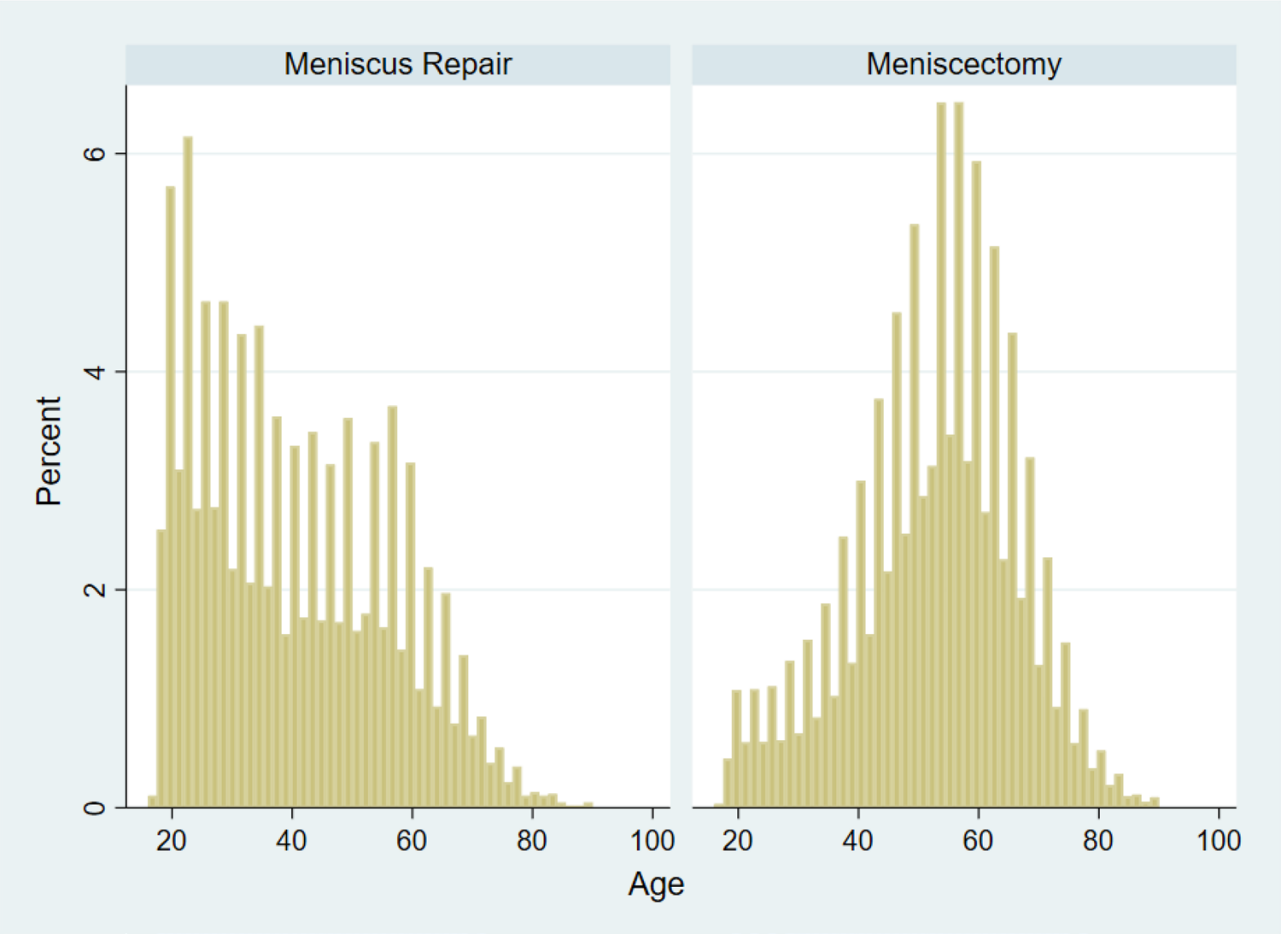
Age distribution by surgery. Age distribution between the meniscus repair and meniscectomy groups demonstrating a trend for meniscus repair in younger patients and meniscectomy in older patients. Mean patient ages were 40.2 ± 15.7 years for the meniscus repair group and 52.6 ± 13.9 years for the meniscectomy group (*p* < 0.001)

Age distribution between the meniscus repair and meniscectomy groups revealed significant differences (Fig. 2). Mean patient ages were 40.2 ± 15.7 years for the meniscus repair group and 52.6 ± 13.9 years for the meniscectomy group (*p* < 0.001). Within the repair group, 46% of patients were over 40 years old, compared to 82% in the meniscectomy group.

### Outcomes and complications

Operative time was significantly longer in the repair group when compared to the meniscectomy group (mean time: 50 vs 30 min, *p* < 0.001). The absolute numbers and rates of all complications are shown in Table 3.

**Table 3:** Thirty-day post-operative complications and outcomes

Complication	Meniscus repair	Meniscectomy	Univariate analysis		Multivariate analysis	
	*n* (%)	*n* (%)	OR (95% CI)	*p* value	Adjusted OR (95% CI)	*p* value
PE	15 (0.2%)	108 (0.1%)	0.4 (0.3–0.8)	0.005	0.3 (0.2–0.6)	< 0.001
VTE	50 (0.8%)	449 (0.4%)	0.6 (0.4–0.8)	< 0.001	0.5 (0.4–0.7)	< 0.001
Wound disruption	0	12 (0.01%)	1		1	
Reoperation	29 (0.5%)	406 (0.4%)	0.9 (0.6–1.3)	(n.s)	0.8 (0.5–1.2)	(n.s)
Readmission	52 (0.9%)	708 (0.8%)	0.9 (0.7–1.2)	(n.s)	0.6 (0.5–0.9)	0.003
SSI						
Superficial	9 (0.1%)	189 (0.2%)	1.3 (0.7–2.6)	(n.s)	1.4 (0.7–2.8)	(n.s)
Deep incisional	4 (0.1%)	60 (0.1%)	0.9 (0.4–2.6)	(n.s)	0.8 (0.3–2.3)	(n.s)

Meniscus repair is the reference group for the univariate and multivariate analysis

All 30-day complication rates were < 1% for both meniscus repair and meniscectomy. Univariate analysis found no significant differences between the meniscus repair and meniscectomy group for superficial SSI (0.1% vs 0.2%, n.s), deep SSI (0.1% vs 0.1%, n.s), reoperation (0.5% vs 0.4%, n.s), and readmission (0.9% vs 0.8%, n.s). In the multivariate logistic regression analysis, which controlled for sex, age, steroid use, and respiratory status, meniscus repair was associated with significantly higher rates of PE, VTE, and readmission compared to meniscectomy: PE (0.2% vs 0.1%, *p* < 0.001), VTE (0.8% vs 0.4%, *p* < 0.001), and readmission (0.9% vs 0.8%, *p* = 0.003). Similar trends and rates were found in patients aged > 40 years undergoing meniscus repair versus meniscectomy. The multivariate analysis revealed that meniscus repair was associated with significantly higher rates of PE (0.4% vs 0.1%, *p* < 0.001), VTE (1.1% vs 0.5%, *p* < 0.001), and all-cause readmission (1.2% vs 0.9%, *p* = 0.01) in patients > 40 years old (Table 4). Complication rates were similarly overall low (< 1.3%) in the > 40 years old group.

**Table 4:** Thirty-day post-operative complications and outcomes in patients > 40 years old

Complication	Meniscus repair (*n* = 2903)	Meniscectomy (*n* = 81,333)	Univariate analysis		Multivariate analysis	
	*n* (%)	*n* (%)	OR (95% CI)	*p* value	Adjusted OR (95%CI)	*p* value
PE	11 (0.4%)	95 (0.1%)	0.3 (0.2–0.6)	< 0.001	0.3 (0.2–0.6)	< 0.001
VTE	31 (1.1%)	373 (0.5%)	0.4 (0.3–0.6)	< 0.001	0.4 (0.3–0.6)	< 0.001
Wound disruption	0 (0.0%)	10 (0.0%)	1		1	
Reoperation	14 (0.5%)	349 (0.4%)	0.9 (0.5–1.5)	(n.s)	0.8 (0.5–1.5)	(n.s)
Readmission	33 (1.2%)	632 (0.9%)	0.7 (0.5–1.0)	(n.s)	0.6 (0.5–0.9)	0.010
SSI						
Superficial	1 (0.0%)	153 (0.2%)	5.5 (0.8–39.1)	(n.s)	5.5 (0.8–39.6)	(n.s)
Deep incisional	3 (0.1%)	47 (0.1%)	0.5 (0.2–1.8)	(n.s)	0.5 (0.2–1.6)	(n.s)

Meniscus repair is the reference group for the univariate and multivariate analysis

## Discussion

The most important finding of the present study was that both arthroscopic meniscus repair and meniscectomy were associated with low short-term complication rates (< 1%). Although arthroscopic meniscus repair was associated with statistically significant increases in 30-day PE, VTE, and readmission rates compared to meniscectomy, similarly low complication rates were seen in the overall (< 1%) and aged > 40-year cohorts (< 1.3%). These findings support meniscus repair over meniscectomy, even in older patients, when repair is possible in the setting of preserved articular cartilage and aids surgeons in the preoperative counselling of patients regarding the risks and benefits of arthroscopic meniscus treatment.

The overall rates of complications across 105,726 patients (< 1%) and among patients aged > 40 years (< 1.3%) are generally consistent with prior reports [3, 10, 21, 22]. Small et al. reported an overall low incidence of complications such as haemarthrosis, infection, and thromboembolic disease following meniscal repair (1.29%) in 310 procedures compared to meniscectomy (1.69%) in 3,617 procedures [21]. Basques et al. also reported an overall 30-day complication rate of 1.17% after meniscectomy, which included stroke/cerebrovascular accident, thromboembolic event, myocardial infarction, renal failure, infections, and reoperation [3]. When comparing short-term 30-day complication rates following meniscus repair directly with meniscectomy, a study of 27,580 patients by Sochacki et al. found that the complication rates after meniscal repair (1.2%) compared with meniscectomy (0.82%) was significantly higher [22]. Additionally, they showed that patients undergoing meniscal repair had higher infection rates (0.4% vs 0.2%) and DVT (0.3% vs 0.1%) compared with those undergoing meniscectomy [22]. These findings are similar to those of this study, except no difference in infection rates between meniscus repair and meniscectomy was seen in this study. The disparity may be attributed to differences in sample size, as the NSQIP cohort included nearly four times the number of patients. Overall, these findings demonstrate that the short-term complications following arthroscopic meniscus repair and meniscectomy both remain low (< 1.3%), suggesting that meniscus repair can be considered whenever possible to maintain meniscus function and optimize load distribution in the knee.

For patients over 40 years old, the meniscal repair group was similarly associated with increased rates of PE, VTE, and readmission compared to meniscectomy; however, 30-day complication rates for meniscal repair were all less than 1.3%. Increasing age is often associated with higher complication rates. Sherman et al. reported in 3,261 arthroscopic knee procedures that patients aged > 50 years were at a higher risk for post-operative infections, haemarthroses, adhesions, cardiovascular issues, and neurological complications [20]. Ozcan et al. also reported an approximately sixfold increase in DVT for patients older than 40 years of age compared with younger patients following knee arthroscopy [17]. As in this study, they found that the overall incidence of DVT was relatively low (0.79%). The authors concluded that this difference may be clinically unimportant due to overall low incidences of DVT in either group [17]. Maletis et al. also observed in 20,770 patients a low 90-day incidence of DVT (0.25%) and PE (0.17%) after elective knee arthroscopy [12]. Furthermore, they found a small but significant increase in incidence of VTE in patients aged ≥ 50 years (0.51%) compared with younger patients (0.34%). In addition to age, medical comorbidities and post-operative immobilization are known risks factors for VTE after lower extremity orthopaedic surgery [2, 3, 14, 17, 20]. Due to increased risks for post-operative complications in older patients with prolonged immobilization, some clinicians may favour performing meniscectomies over meniscal repairs in these patients to allow for early weightbearing. Despite these concerns, this study demonstrated that both arthroscopic meniscus repair and meniscectomy have low rates of post-operative 30-day complications (< 1.3%) including VTE, PE, infection, readmission, and reoperation in patients > 40 years old. These results suggest that meniscus repair should be performed when feasible for patients > 40 years old and may help surgeons during the preoperative counselling process.

NSQIP has uniform standards for the reporting of adverse events and undergoes inter-reliability audits that make it a highly reliable resource. Nevertheless, the data can be subject to errors in coding and underreporting. The retrospective analyses are dependent upon the accuracy of the CPT codes reported, and inaccuracies in miscoding or noncoding by physicians are potential sources of error. Second, patients are only tracked during the first 30 days after surgery, and the database does not report orthopaedic-specific complications. Although the first 30 days are an important early post-operative period, studies that track longer-term outcomes may yield a more comprehensive analysis. Further, since the data in the current study were from multiple institutions, there is likely heterogeneity in practice environment, sterilization technique, and surgical indications that may influence the results. Post-operative rehabilitation protocols, including weightbearing status, range of motion restrictions, and DVT prophylaxis, may have differed between groups as well as among patients treated with meniscus repair. Last, this study’s observational nonrandomized nature limits the external validity of our results due to potential selection bias. Despite these limitations, this database study benefits from a large number of patients and high-quality data collection process in identifying the short-term complications rates following meniscal repair and meniscectomy.

In the present study, both arthroscopic meniscus repair and meniscectomy were associated with low short-term complication rates, including in patients over the age of 40 years (< 1.3%). For surgeons, these findings support meniscus repair over meniscectomy when repair is possible, even in older patients, in the setting of preserved articular cartilage.

## Conclusion

Arthroscopic meniscus repair and meniscectomy are both low-risk procedures with 30-day complication rates < 1% overall and < 1.3% among patients aged > 40 years. These findings support meniscus repair whenever feasible, even in older patients, in the setting of preserved articular cartilage. Understanding of the short-term complication rates after arthroscopic meniscus repair and meniscectomy can aid surgeons in providing comprehensive preoperative counselling to patients considering such treatments, specifically when discussing the risks and benefits of meniscus repair.

## Data Availability

The data that support the findings of this study are available from the authors but restrictions apply to the availability of these data, which is limited to ACS NSQIP participants, for the current study, and so are not publicly available. Data are, however, available from the authors upon reasonable request.

## References

[1] Abrams GD, Frank RM, Gupta AK, Harris JD, McCormick FM, Cole BJ (2013). Trends in meniscus repair and meniscectomy in the United States, 2005–2011. Am J Sports Med.

[2] Abram SGF, Judge A, Beard DJ, Price AJ (2018). Adverse outcomes after arthroscopic partial meniscectomy: a study of 700 000 procedures in the National Hospital Episode Statistics database for England. Lancet.

[3] Basques BA, Gardner EC, Varthi AG (2014). Risk factors for short-term adverse events and readmission after arthroscopic meniscectomy. Am J Sports Med.

[4] Beamer BS, Walley KC, Okajima S (2017). Changes in contact area in meniscus horizontal cleavage tears subjected to repair and resection. Arthroscopy.

[5] Cohen ME, Dimick JB, Bilimoria KY, Ko CY, Richards K, Hall BL (2009). Risk adjustment in the American College of Surgeons National Surgical Quality Improvement Program: a comparison of logistic versus hierarchical modeling. J Am Coll Surg.

[6] Eberbach H, Zwingmann J, Hohloch L (2017). Sport-specific outcomes after isolated meniscal repair: a systematic review. Knee Surg Sport Traumatol Arthrosc.

[7] Hall MJ, Lawrence L (1998). Ambulatory surgery in the United States, 1996. Adv Data.

[8] Hirose T, Mae T, Ogasawara I (2022). Meniscal displacement and loss of load-transmission function after radial tear of the lateral meniscus in a porcine model: new insights into the functional dynamics of the injured meniscus. Am J Sports Med.

[9] Husen M, Kennedy NI, Till S et al (2022) Benefits of meniscal repair in selected patients aged 60 years and older. Orthop J Sports Med 10(9):2325967 10.1177/23259671221117491 PMC9445464 36081411

[10] Jones JC, Burks R, Owens BD, Sturdivant RX, Svoboda SJ, Cameron KL (2012). Incidence and risk factors associated with meniscal injuries among active-duty US military service members. J Athl Train.

[11] LaPrade CM, Jansson KS, Dornan G, Smith SD, Wijdicks CA, LaPrade RF (2014). Altered tibiofemoral contact mechanics due to lateral meniscus posterior horn root avulsions and radial tears can be restored with in situ pull-out suture repairs. J Bone Jt Surg.

[12] Maletis GB, Inacio MCS, Reynolds S, Funahashi TT (2012). Incidence of symptomatic venous thromboembolism after elective knee arthroscopy. J Bone Jt Surg.

[13] Martin CT, Pugely AJ, Gao Y, Wolf BR (2013) Risk factors for thirty-day morbidity and mortality following knee arthroscopy: a review of 12,271 patients from the national surgical quality improvement program database. J Bone Jt Surg 95(14):1–10 10.2106/JBJS.L.01440 23864189

[14] Mizel MS, Thomas HT, Michelson JD (1998). Thromboembolism after foot and ankle surgery a multicenter study. Clin Orthop Relat Res.

[15] Muriuki MG, Tuason DA, Tucker BG, Harner CD (2011). Changes in tibiofemoral contact mechanics following radial split and vertical tears of the medial meniscus: an in vitro investigation of the efficacy of arthroscopic repair. J Bone Jt Surg.

[16] Ode GE, Van Thiel GS, McArthur SA (2012). Effects of serial sectioning and repair of radial tears in the lateral meniscus. Am J Sports Med.

[17] Özcan M, Erem M, Turan FN (2019) Symptomatic deep vein thrombosis following elective knee arthroscopy over the age of 40. Clin Appl Thromb Hemost 25:1076 10.1177/1076029619852167 PMC6714953 31115250

[18] Parker BR, Hurwitz S, Spang J, Creighton R, Kamath G (2016). Surgical trends in the treatment of meniscal tears. Am J Sports Med.

[19] Paxton ES, Stock MV, Brophy RH (2011). Meniscal repair versus partial meniscectomy: a systematic review comparing reoperation rates and clinical outcomes. Arthroscopy.

[20] Sherman OH, Fox JM, Snyder SJ, Del Pizzo W, Friedman MJ, Ferkel RD LM (1986) Arthroscopy—“no-problem surgery”. An analysis of complications in two thousand six hundred and forty cases. J Bone Jt Surg 68(2):256–265 3753706

[21] Small NC (1988). Complications in arthroscopic surgery performed by experienced arthroscopists. Arthroscopy.

[22] Sochacki KR, Varshneya K, Calcei JG (2020). Comparing meniscectomy and meniscal repair: a matched cohort analysis utilizing a National Insurance Database. Am J Sports Med.

[23] Stein T, Mehling AP, Welsch F, Von Eisenhart-Rothe R, Jäger A (2010). Long-term outcome after arthroscopic meniscal repair versus arthroscopic partial meniscectomy for traumatic meniscal tears. Am J Sports Med.

[24] von Elm E, Altman DG, Egger M, Pocock SJ, Gøtzsche PC, Vandenbroucke JP (2008). The Strengthening the Reporting of Observational Studies in Epidemiology (STROBE) statement: guidelines for reporting observational studies. J Clin Epidemiol.

